# Strengthening national preparedness for smallpox: an update.

**DOI:** 10.3201/eid0701.700155

**Published:** 2001

**Authors:** J W LeDuc, P B Jahrling

**Affiliations:** Centers for Disease Control and Prevention, Atlanta, Georgia, USA.


### Strengthening National Preparedness for Smallpox: An Update

Concern that smallpox virus may be used as a biological weapon of mass destruction has prompted calls for production of additional vaccine and new research into variola virus diagnostics and clinical interventions. Only 15.4 million doses of smallpox vaccine, produced approximately 20 years ago, exist in the United States (1). While virtually all lots remain potent, additional vaccine would clearly be needed in a national emergency involving smallpox virus. Global eradication of natural smallpox disease was declared in 1980; with eradication, most research activities involving the virus ended. Although the complete genomic sequence of selected isolates of variola virus is known (2), the diagnosis and treatment of smallpox infection have not changed in the past two decades. Recognizing the need for advancement in these areas before variola virus stocks are destroyed, the World Health Organization (WHO) passed a resolution (WHA 52.10) in 1999 extending the date of destruction of all remaining variola virus stocks until the end of 2002. The midpoint of this period is an appropriate time to review progress made in vaccine production and variola virus research and to outline the next steps.

### Vaccine Production

On September 20, 2000, the Centers for Disease Control and Prevention (CDC) entered into an agreement with OraVax (Cambridge, MA) to produce a new smallpox vaccine. Like the vaccine used to eradicate smallpox, the new vaccine will contain live vaccinia virus; however, it will be produced in cell cultures by modern vaccine production techniques. OraVax will coordinate full clinical testing of the vaccine and submit a licensing application to the U.S. Food and Drug Administration (FDA) for the prevention of smallpox in adults and children. Forty million doses of the new vaccine will be produced initially, with anticipated delivery of the first full-scale production lots in 2004. The agreement calls for sustained annual production through 2020 to replace outdated vaccine and allows for increased production should an emergency arise. The vaccine will be administered with bifurcated needles (also produced by OraVax), which create a localized vaccine “pock” and confer protective immunity. The vaccine will be held in reserve as part of the national stockpile and be released only in the event of a confirmed case of smallpox or when vaccination against vaccinia virus is warranted. The agreement allows OraVax to produce additional vaccine for other markets, including international buyers.

### Variola Virus Research

A research plan, implemented at CDC by scientists from both the Department of Defense and CDC and including extensive collaborations with scientists from the National Institutes of Health and other organizations, is being undertaken with WHO concurrence. All work with live variola virus is done under biosafety level 4 containment conditions at CDC. Smallpox virus is officially retained at only two facilities in the world: at CDC in the United States and the State Research Center of Virology and Biotechnology in Novosibirsk, Russia. Research teams from both institutions are coordinating activities to avoid duplication and gain the maximum amount of information possible before final destruction of the virus.

#### Strain Evaluation

Of 461 isolates in the smallpox virus collection at CDC, 49 were selected for further characterization. These isolates, which included both variola major and variola minor, were selected to represent the greatest diversity in date of collection and geographic region. Of the 49 isolates tested for viability, 45 were successfully recovered, and seed stocks were prepared for subsequent studies. This group of 45 represented isolates from as early as 1939 and as late as the 1970s; all major geographic regions were represented. Study of these isolates is based on three research themes: application of modern serologic and genomic methods in the diagnosis of variola virus disease; determination of candidate antiviral drug activity against this virus; and investigation of the pathogenesis of smallpox infection, especially through the development of a nonhuman primate model to replicate human smallpox infection. The research team carefully outlined all experimental work to be undertaken with variola virus, incorporating suggestions from a peer group of highly qualified external experts from academia and industry; the first set of experiments was conducted from January to July 2000 in the CDC maximum containment laboratory.

### Serologic Assays

Because enzyme immunoassay technology was still in its infancy when smallpox was eradicated, during the first series of experiments, polyclonal and monoclonal antibodies had to be produced for developing enzyme-linked immunosorbent assays to measure variola virus-specific immunoglobulin (Ig) M, IgG, and antigen. These reagents are now being evaluated by prototype assays with inactivated viral antigens. This work will continue for the foreseeable future.

### Nucleic Acid-Based Diagnostics

Viral DNA was extracted from all 45 successfully recovered isolates, was purified and inactivated, and is now being examined by restriction fragment-length polymorphism developed by an extended polymerase chain reaction assay that amplifies viral genome into 20 overlapping products of approximately 10 kilobases each. These products cover virtually the entire length of the viral genome and include sequences in essential genes and genes likely needed for pathogenesis. Preliminary results indicate that the data thus generated offer a good low-resolution overview of genetic diversity of variola viruses and are being used to differentiate strains, infer phylogeny, and identify as many as 10 additional variola isolates for complete genome sequencing. Two isolates, Somalia 77 and Congo 70, were specifically suggested by WHO for sequencing, and this work has begun. A dedicated sequence and bioinformatics facility being developed at CDC will be used to undertake this effort and to begin constructing a genomic signature database, not only for smallpox but also, over time, for other pathogens with bioterrorism potential.

### Antiviral Drugs

Two hundred seventy-four antiviral drug compounds were screened for activity and therapeutic indices against variola, monkeypox, cowpox, camelpox, and vaccinia viruses by two cell culture assays. Many of these compounds were provided for testing under collaborative arrangements facilitated by an orthopox antiviral research initiative of the National Institute of Allergy and Infectious Diseases. Previous studies identified a nucleoside phosphonate DNA polymerase inhibitor, cidofovir (Vistide), as being active against poxviruses, including variola. In the current trial, cidofovir and its prodrug (cyclic HPMPC) were evaluated against 31 strains of variola, which were selected to cover a wide geographic area and time span. No substantial differences in inhibition among strains were observed, which suggests that cidofovir-resistant strains are unlikely. The in vitro inhibition was further characterized in multiple cell lines to meet FDA requirements. However, another class of antiviral drugs, the *S*-adenosylhomocysteine hydrolase inhibitors, showed considerable variation in the 50% inhibitory dose between variola isolates; this effect should be investigated further.

Two approaches to the development of an oral prodrug of cidofovir yielded compounds with improved antiviral activity. In addition, the current series of experiments identified 27 other compounds, including completely new classes of drugs, that appear to be active against variola and other orthopoxviruses. In fact, 10 compounds had therapeutic indices greater than 200, while cidofovir had indices greater than 10; 3 compounds had therapeutic indices greater than 1,500. When work resumes in early 2001 with live variola virus, we will continue to evaluate these and additional compounds for activity, including analogs designed for oral administration. The most promising compounds emerging from this in vitro testing will be evaluated in animal models, e.g., cowpox and vaccinia in mice and eventually monkeypox virus challenge in nonhuman primates. All promising compounds will be tested against a battery of surrogate orthopox viruses to guide evaluation of new antiviral compounds after variola virus is no longer available.

### Animal Models

A major goal of the current research is to define an animal model that faithfully replicates human smallpox. Such a model would be extremely valuable in evaluating candidate antiviral drugs and novel diagnostic assays and in defining the pathogenesis of smallpox. Consequently, two groups of four cynomolgus macaques were exposed to two variola virus strains at a high dose ($>10^8$ PFU) by the aerosol route. Clear evidence of infection was found; the animals had transient fevers, perturbations in cytokine titers in serum, and mild exanthemous lesions. A few of the monkeys showed signs of bronchopneumonia, but none died or had disease similar to the classic smallpox seen in humans. Another series of experiments will be undertaken with different variola isolates to confirm these preliminary observations and generate additional clinical material to validate the diagnostic assays under development.

The results of the research now under way, coupled with the promise of renewed production of smallpox vaccine, will better prepare the United States—and indeed the entire world—for the possibility that smallpox virus might be used as a terrorist weapon of mass destruction.

**James W. LeDuc* and Peter B. Jahrling†**
*Centers for Disease Control and Prevention, Atlanta, Georgia, USA; †United States Army Medical Research Institute of Infectious Diseases, Fort Detrick, Frederick, Maryland, USA

### References

1. LeDuc JW, Becher J. Current status of smallpox vaccine. Emerg Infect Dis 1999;5:593-4.
2. Shchelkunov SN, Massung RF, Esposito JJ. Comparison of the genome DNA sequences of Bangladesh-1975 and India-1967 variola viruses. Virus Res 1995;36:107-18.
